# The African National Congress’s factionalism and targeted killings as risks to human security in KwaZulu-Natal province

**DOI:** 10.4102/jamba.v16i1.1502

**Published:** 2024-04-26

**Authors:** Collin O. Mongale, Jan C. Venter

**Affiliations:** 1 Department of Political Studies and International Relations, Faculty of Humanities, North-West University, Potchefstroom, South Africa

**Keywords:** African National Congress (ANC), factionalism, human security, Inkatha Freedom Party (IFP), KwaZulu-Natal, political assassinations, targeted killings

## Abstract

A prevalence of political violence and political assassinations characterised post-1994 South Africa. These politically motivated killings appeared to be dominant in the controversial KwaZulu-Natal (KZN) province. Political killings in South Africa started as a form of inter-party warfare, especially during the transition to democracy, when the two rivals, the African National Congress (ANC) and the Inkatha Freedom Party (IFP), fought each other for some areas of Gauteng and KwaZulu-Natal provinces. However, following the dominance of the ANC in the KZN Province, members of the ruling party fought each other for positions in government and political party structures. Considering this, the article analyses the crisis of factionalism by examining the ANC’s intra-party tensions and targeted killings, and how this poses a risk to human security in KZN. Methodologically, the article employs a qualitative literature assessment and content analysis is used to delve into the impact of intra-party tensions and targeted killings on human security in the KZN province.

**Contribution:**

In quest for curbing the crisis of factionalism in the ruling ANC, the article recommends that the ANC needs to re-visit its leadership selection as these killings have seemingly happened during leadership selection, which leads to ruthless competition of positions in government and party structures. Members of the ruling party need to identify themselves as one, as opposed to belonging to different factional groups within the party. Failure by the ruling party to address divisions within the organisation shall result in more fatal killings resulting from competition for positions and resources.

## Introduction

The concept of ‘factionalism’ comprises different interpretations, ranging from a neutral assessment of different centres of power blocs within a party or organisation to a value-laden interpretation that links factionalism with patronage, self-interest and often with self-enrichment (Mukwedeya 2015:119). Factions are considered as a significant aspect of political competition (eds. Belloni & Beller 1978:13). Moreover, Zariski (1960) postulates that factions are an important structural feature inside a political party:

[*W*]e might define a faction as any intra-party combination, clique, or grouping whose members share a sense of common identity and common purpose and are organized to act collectively – as a distinct bloc within the party – to achieve their goals. These goals may include any, several, or all the following: patronage (control of party and government office by members of the faction), the fulfilment of local, regional, or group interests, influence on party strategy, influence on party and governmental policy, and the promotion of a discrete set of values to which members of the faction subscribe (Zariski 1960:33 in Broucek 2009:468).

Based on this definition provided by Zariski, a faction refers to a group that exists within a larger group – and, in the context of this article, a political party such as the African National Congress (ANC) (Mukwedeya 2015:120). Factionalism is regarded by Mukwedeya (2015:118) as a process of partitioning a political party into sub-units that tend to engage in collective action to attain their members’ objectives and interests (Broucek 2009). Against this backdrop, this article investigates the crisis of factionalism: ANC intra-party tensions and targeted killings as a risk to human security in KwaZulu-Natal (KZN) Province. The standpoint of the article is that factionalism within the ANC in KZN has resulted in intra-party violence and the targeted killings of members of the same political party who compete to gain access to state resources. This being the case, factionalism in the ANC poses a risk to the security of people, as the acts of violence and killings have resulted in loss of life and post-traumatic stress disorder, including poverty (by killing a family breadwinner) (Mongale 2022b:66). This article is designed to address the following objectives: firstly, to analyse the nature and dynamics of factionalism in the African National Congress and secondly, to analyse how intra-party violence and targeted killings serve as risks to human security in the province.

## Methodology

Methodologically, this article adopted a qualitative literature assessment, based on secondary sources. As such, the existing textual material produced by various scholars on factionalism and targeted killings in KZN will be used to achieve the objectives of this study. Qualitative research methodology entails the collection and analysis of non-statistical data such as text, video or audio to understand different ideas, opinions or experiences (Nieuwenhuis 2016:50). At times, qualitative research can be used to gain in-depth insights into a particular problem or produce new ideas for research; thus, assisting scholars to identify the gaps that exist in the literature. Commonly, this method of research is used in the Humanities and Social Sciences, in subjects related to political studies, sociology, history, anthropology and health sciences. This method remains relevant in this article because the study will utilise scholarly literature written by other scholars about intra-party tensions and institutionalised killings among ANC members in KZN.

## Conceptual framework: Human security perspective

The concept of human security places its focus on the individual as the main referent object of security (Acharya 2007:52). As such, human security is about the security of the people, rather than of states or governments as it was the case in traditional security. Whereby security included mainly the protection of the state against external threats or invasion (Kaldor 2013). Advocates of human security such as Alkire (2003), Acharya (2007), and Tadjbakhsh and Chenoy (2007) find it to be a vital step forward in indicating possible threats and dangers to human safety and survival, which are caused by poverty, disease, environmental stress, human rights violations and armed conflict. Furthermore, Singh (2008:167) defines the concept of human security as comprising two main aspects: Firstly, it refers to safety from such chronic threats as hunger, disease and repression. Secondly, it means protection from unforeseen and harmful disruptions that tamper with the pattern of daily life, whether in homes, jobs or communities (UNDP 1994). According to Alkire (2003:3), human security is derived from the protection of human life; hence, the protection of a human being serves as a vital aspect of human security. Furthermore, human security can be based on a multi-sectoral understanding of possible threats and dangers that can lead to insecurities. In Table 1, the article highlights that the notion of human security entails different types of security, which are also supported by examples of the main threats to these types of security.

**TABLE 1:** Possible types of human security and examples of main threats.

Types of security	Examples of main threats
Economic security	Persistent poverty and unemployment
Food security	Hunger and famine
Health security	Deadly infectious diseases, unsafe food, malnutrition and lack of access to basic health care
Personal security	Physical violence, crime, terrorism, domestic violence and child labour
Community security	Inter-ethnic, religious and other identity-based tensions
Political security	Political repression and human rights abuses

*Source:* United Nations Trust Fund for Human Security (UNTFHS), 2016, *Human security handbook. An Integrated approach for the realization of the development sustainable goals and the priority areas of the International Community and the United Nations System.*

The concept of human security also reflects upon the existing interconnectedness between the threats and responses while addressing these insecurities (Alkire 2003:27) because human security threats are mutually reinforcing and interconnected in two ways (UNTFHS 2016:7). For example, human security threats are interlinked in a domino effect, which means, each threat feeds on the other (Kaldor 2007). As Alkire (2003:27) had alluded before, the prevalence of violent conflicts could lead to relative deprivation and poverty, which in turn could result in the depletion of resources, infectious diseases and education deficits. Because of spillover effects, these domestic threats can also spread widely across the region, thus, leading to negative externalities for regional and international security (UNTFHS 2016:6). In essence, the UNTFHS Report (2016:6) stated that the human security approach provided a new way of thinking about challenges that continue to hamper the global community.

## The nature and dynamics of factionalism in the African National Congress

In South Africa, the ANC faces factionalism, which has become a defining characteristic of the ANC’s intra-party politics, primarily during the lead up to and after the 2007 National Elective Conference held in Polokwane (Mukwedeya 2015:117). The Polokwane Conference was a turning point in the post-apartheid ANC history because the party was heavily divided along factional lines (Venter & Duvenhage 2008:629). In turn, these divisions within the party led to a vicious contestation of power and leadership positions (Mongale 2022a:12). In addition, tribalism served as a basis for gathering support ahead of the Polokwane Conference (Mukwedeya 2015:118). For one, in 2007, ahead of the Polokwane Conference, Jacob Zuma used his Zulu background to garner support in KZN (i.e. Zuma’s stronghold). Thus, Zuma emerged as the victor at the Polokwane Conference (Gumede 2016). Moreover, Zuma in his campaign to be victorious at the Polokwane Conference portrayed any opposition to his contestation to power as an ‘anti-Zulu’ conspiracy (Chikane 2012). Labelling Thabo Mbeki as a member of an educated elite who had declared ‘war’ on the traditions of Africa, its institutions and its style of leadership (Mongale 2022b:62). Against this backdrop, during Zuma’s political campaign, his supporters wore ‘100% Zulu’ T-shirts, an indication that Zuma was in pursuit of the politics of ethnic patronage (Gumede 2016).

The election of Zuma as the ANC president in 2007, including his re-election at the Mangaung Conference in 2012, symbolised the triumph of the so-called conservative wing of Zulu nationalism (Gumede 2016). This victory also led to the retreat of the progressives. As a result of Zuma’s presidency, many individuals from KZN were appointed to key positions, both in the government and in the ANC, more especially in the security networks such as the appointment of Thulani Dlodlo who was allegedly accused of turning the State Security unit into a factional structure, which is bound to protect former president Jacob Zuma and his political faction (Mvumvu 2020). This situation led critics to warn of the danger of the emerging ‘Zulufication’ of the ruling ANC (Gumede 2016).

In this regard, Mongale (2022b:62) takes the position that issues of tribe-enabled factionalism and contestation for power in the ANC are seen in situations whereby a person’s place of origin serves as a determining factor of where one is likely to gain political support. In 2014, Thabo Mbeki stated that in the President’s Ministry, for example, when a minister originated from a certain region, so would the officials in that department (Mongale 2022b:62). Mbeki termed this practice the ‘homeboy’ phenomenon. In his concluding remarks, Mbeki alluded that tribalism was ‘raising its head again’ in post-apartheid South Africa (Gumede 2016). In addition, Mbeki also applied the same approach during his presidency. As an isiXhosa-speaker by birth, Mbeki was accused of surrounding himself with a group of key individuals who originated from the Eastern Cape, what was proclaimed to be ‘Xhosa-Nostra’ (Gumede 2016).

Factionalism in the ANC could be also linked to the events of September 2005, when Jacob Zuma was sacked as the deputy president by President Thabo Mbeki. This came after Zuma’s financial adviser, Schabir Schaik, was convicted of fraud and corruption by the Durban High Court (Chikane 2007:58). The sacking of Zuma from his deputy president position instigated a coming together of forces, which had increasingly become alienated by the leadership of Mbeki (Mongale 2022b:62). Zuma assumed the leadership position of the ANC after defeating Mbeki at the Polokwane Conference in December 2007 (Mukwedeya 2015:119).

The events of the Polokwane and Mangaung Conferences would trickle down to engulf the ANC’s provincial, district, and regional structures in purges of supporters of the then party’s president, Thabo Mbeki (Mukwedeya 2015:119). The results of these purges of Mbeki supporters led to municipalities becoming battlegrounds; this was witnessed through the numerous power struggles that existed between ANC executive committee members, councillors, mayors, municipal managers and other bureaucrats (Mongale 2022b:63).

Factionalism increased in the ANC before and after the 2017 National Elective Conference hosted at the NASREC Conference Centre in Soweto (BusinessTech 2016). At NASREC, Jacob Zuma, who was stepping down as the party leader after two terms, backed senior ANC politician, the former AU Chairperson and his ex-wife, Nkosazana Dlamini-Zuma, against Cyril Ramaphosa who emerged as the victor with 52% of the votes (Hunter 2017). The win by Ramaphosa at NASREC exacerbated factions in the ANC because the party was split by camps loyal to President Ramaphosa, who referred to as the CR-17 faction, erstwhile rival Nkosazana Dlamini-Zuma’s faction, NDZ, as well as those who aligned to the former president Jacob Zuma (Mongale 2022a:12). The nature of factionalism in the ruling ANC and contestation for power has brought back the battles of the inxiles and exiles, which has plagued the party since democratic transition. For instance, those who served the party in exile during the struggle against apartheid have become prone to undermine Ramaphosa’s leadership (Nkanjeni 2021). For instance, following the July 2021 riots in KZN and Gauteng, the Minister of Deference Mapisa-Nqakula was accused of ignoring early warning about the planned insurrection as she was quoted saying:

I am being portrayed as an irresponsible young girl. I think I’m senior enough, I’m politically mature enough, to appreciate that there are things you cannot play around with, one of those is this situation we are finding ourselves in. (Nkanjeni 2021)

Moreover, Mapisa Nqakula went as far as reciting her struggle credentials that included being a member of the ANC’s military wing uMkhonto we Sizwe (MK) and her time in exile. During the same time, she was supported by those who sympathised with the suspended ANC secretary-general Ace Magashule, who is a staunch supporter of Jacob Zuma and his Radical Economic Transformation (RET) faction (Mongale 2022a:12). Those who went to exile perceive themselves as seniorities to those who never went to exile during the liberation struggle; henceforth, they continue to undermine the presidency of Cyril Ramaphosa who holds no military training and never served the ANC in exile during the days of apartheid in South Africa.

In respect of factionalism in KZN, Edwin Mkhize (who served as the provincial secretary of COSATU in KZN) asserted that there was a link between political killings in the province and ANC conferences (Mongale 2022b:62). Mkhize, therefore, alleged that the intensifying political violence and killings in the province could be blamed on issues of corruption, maladministration and the ensuing factionalism in the ANC. The issue of ANC conferences and killings was explained by Krelekrele (2018:4), who highlighted the killing of Lungisani Nguni, the ANC’s former branch chairperson in KZN. Nguni was shot and killed in broad daylight in November 2017, while he was delivering items for a school nutrition programme. Allegations are that, his assassination was linked to the conference that he was supposed to attend as a delegate in December 2017 (Krelekrele 2018:5). The horror seemed far from over. For example, in Howick in April 2018, where the ANC had a branch meeting, bodyguards assigned to the ANC leaders drew their guns, showing their readiness to shoot after a meeting resulted in chaos and disruption (Wicks 2018).

The chaos was instigated by a group of branch members in the Moses Mabhida Region, who insisted that they had been sidelined from attending the meeting, and they stormed the gates while the meeting was being held (Wicks 2018). The meeting had been called to brief branch chairpersons on their readiness to elect new regional and provincial leadership, following the dissolving of the provincial executive committee by the Pietermaritzburg High Court in January 2018 (Mongale 2022b:63). This was performed under the premise of curbing the violent nature of political killings, which was exacerbated by ANC conferences, as members of the ruling party would eliminate each other through political killings as a means of increasing their chance of being elected in the party conferences (Bruce 2016).

Mark Shaw, a criminologist from the University of Cape Town, published a book about the use of hitmen in South Africa titled *Hitmen for Hire, exposing South Africa’s underworld* (Shaw 2017). In his narrative, Shaw (2017:33) asserts that there has been an increase in the number of assassinations over the years. ‘Our most recent data suggests that the increase is because of greater political tensions’. According to Shaw (2017:43), professional hits remained complex to identify, as they were often disguised as accidents or as incidents of crime.

The South African Local Government Association (SALGA) report that examined violence in local municipalities indicated that intra-party contestation for power was believed to be an appropriate reason for violence (SALGA 2019:13). There was a perceived prevalence of strong competition for positions within ANC’s structures exacerbated by the alleged opportunities attached to the positions (Bruce 2013:4).

In addition, holding political office in local or provincial structures provided access to resources, the furthering of business interests and personal enrichment (SALGA 2019:13). Moreover, the existing poor socio-economic conditions tended to influence competition for positions (factionalism) as a means of accessing control to socio-political and economic resources (Bruce 2013:3). Consequently, political positions are highly contested, which instigates political violence perpetuated by different political parties or individuals within the party, predominantly the ANC, which is dominant in KZN’s political landscape (De Haas 2016:46).

In KZN, threats also existed that were associated with intra-party factions during local government elections (SALGA 2019:28). These threats and factions arose because of different candidates who perceived positions in local government as a means of accessing resources to further their own selfish interests (Mongale 2022b:63). According to De Haas (2016:44), there had been an increase in the manipulation of party lists, which resulted in violence between different factions. In KZN, such factionalism led to the intimidation and assassination of various ward councillors; therefore, these actions undermined South Africa’s electoral democracy (SALGA 2019:28).

## Findings and analysis

### The role of decentralisation in the fight for resources

The politics of the KZN Province are heavily characterised by competition for resources stemming from factions, in most cases (De Haas 2016:43), which often results in acts of corruption. Duncan (2010) argues that corruption can be associated with the devolution of state responsibilities, which tends to perpetuate the outsourcing of state responsibilities; this occurred because of the decentralisation of the state’s powers. Decentralisation, as defined by Siddle and Koelble (2015), is a process through which powers, functions, responsibilities and resources are relocated from the central government to the local government, as well as to other decentralised entities.

In relation to this article, an important component of decentralisation that is applicable in this context is political decentralisation (Stanton 2009). Political decentralisation refers to the process of delegating political power to subnational state actors such as ward councillors (Stanton 2009). In post-apartheid South Africa, the devolution of government and state power was accompanied by an increase in the number of local government-level politicians and senior government officials vested with the responsibility of managing resources (SALGA 2019:24).

Such decentralization of power to other sub-national state institutions to control their resources by different groups in society representing different interests (Bruce 2014b:3). There is the contestation for political office as a means of monopolising state resources to accumulate resources for personal benefit (Calderon et al. 2018). The contestation for resources by political leaders has led to the political killings of ward councillors in KZN (Mongale 2022b:72).

The assertions made by Mongale (2021:163) are that the competition for political office and political resources triggers most political killings in South Africa. In this regard, there is a need to make the link between power distribution model and political violence, which often results in killings executed for political means (Kramer 2020:25). Financial decentralisation delegates fiscal power and authority to local government structures (SALGA 2019:22). Furthermore, there is a link between fiscal power decentralisation, outsourcing and competition for resources, because some of government duties such as rendering services are being extended to third parties through tenders and contracts. Often at times, these tenders and government contracts are awarded through patronage networks and factional lines (Ardé 2021:122–125. This occurs because of the neoliberal system that privatises social services, resulting in competing interests from different groups, which serves as a breeding ground for political violence and killings in the fight for power and ownership, or the control of resources (De Haas 2016:53). It is in this regard that Bruce (2014) and De Haas (2016) asserted that business interests were inseparable from many political killings in KZN because many people were executed to protect the financial interests of political leaders at local government level (Bruce 2014a; De Haas 2016).

Krelekrele (2019:3) observed that in KZN, there is an overlap between killings directly linked to political rivalry and killings that result from a fight over resources. In other instances, they are the same thing. For example, Mongale (2021:160) argued that many taxi owners in the province fight for routes, transport tenders and other businesses related to the taxi industry. The *modus operandi* employed by taxi owners is that, they tend to hire hitmen to deal with their rivals (Thomas 2018:32). According to Krelekrele (2019:3), some taxi owners are politically involved. At the nucleus of all the violence in KZN, there is a culture of impunity (Thomas 2018:32). This is encouraged by the lack of political will to deal with transgressions by taxi owners and people in their circle (Thomas 2018:33).

Thomas (2018), De Haas (2016) and Krelekrele (2019) highlighted that often taxi owners employ well-trained gunmen, whom they place to work as bodyguards for politicians, and there is limited control over these bodyguards. According to Hlengiwe Mkhaliphi of the Economic Freedom Fighters (EFF), ‘There is no political law taking place in KZN. Therefore, what is happening when we see people are killed is the factions that is taking place within the ANC’. The fight is primarily about resources: ‘One faction wants to eat’ (Mongale 2022b:79). As such, Hlengiwe dismissed the notion of ‘political killings’ in the province and postulated that there was a war within the ANC itself (Ardé 2020:18–25). In his testimony before the Moerane Commission of Enquiry (2018), the then EFF Spokesperson Mbuyiseni Ndlozi argued that the prevalence of killings in KZN was influenced by criminality, as factions in the ANC fought for tenders (Krelekrele 2019:3). ‘There is no politics here. They are killing each other the same as a thug would come to your house and kill you for your cell phone’. Ndlozi added, ‘To brand it political is to almost suspend a due police investigation that must take place’ (Moerane Commission 2018:290).

The EFF further contended that what was unfolding in the province was the ANC fighting for resources among themselves – that was the root cause of the violence, and in particular, the issue of who got to control the multi-billion budget of the eThekwini Metro Municipality (Krelekrele 2019:4). In the 2009–2010 report by the Auditor General Terence Nombembe, it was stated that the eThekwini municipality had irregularly spent R532 million, with 10 councillors having interests in entities that were awarded government contracts (Maharaj 2020). The high profile case of corruption in eThekwini municipality was reported in May 2019, whereby the city Mayor Zandile Gumede, councillor Mondli Mthembu and Robert Abbu, and Deputy Head of strategic and New Development in the eThekwini municipality were arrested after facing the allegation of corruption of over R389 million tender for the Durban Solid Waste project (Maharaj 2020). Nevertheless, factions that existed within the ANC led to the fight for resources and ultimately served as the root cause of the killings in the province (Mongale 2021:160). The intra-party conflicts did not denote any form of a battle of ideologies, but rather a marathon between factions characterised by the ‘my turn to eat’ syndrome (Mongale 2021:161).

### Intra-party tensions and targeted killings as risks to human security

Scholarly literature on political violence, including political killings in South Africa, has focused heavily on inter-party political violence (Selinger 2016; Simpson et al. 2018:03; Taylor 2002). In South Africa, this is evident in the historic political violence and killings resulting from interparty dynamics, employed as a means of eliminating any rivals (Von Holt 2014). Some literature has revealed developments in terms of the way political violence unfolds; this includes studies by Bruce (2009, 2014) and De Haas (2016) who indicated that political violence mixed with political killings in KZN were driven by intra-party dynamics. According to SALGA (2019:28), the underlying reason behind intra-party political violence and political killings is the contestation over political power. Bruce (2009, 2014) asserted that the key motive behind these killings was related to the value attached to holding public office.

This tendency of competition for political office was evident in the political killings that unfolded during the 2016 local government elections (Sempijja & Mongale 2022:465) and the national elections, which were held in 2019. Thomas (2021:14), De Haas (2016), Bruce (2013), Krelekrele (2019) and Shaw (2019) argue that the contestation for political office is exacerbated by the lack of qualifications among many politicians, which contributes to unemployment and thus serve as a threat to economic security as indicated in Table 1. Therefore, compelling politicians to be protective of their positions and their political landscape to the extent of eliminating their competitors. Consequently, tampering with other people’s basic human rights such as a right to life (Constitution of the Republic of South Africa, Act 108 of 1996) while posing a threat to political security such as political repression and human rights abuses as indicated in Table 1. Perhaps, it was argued that politics, crime and corruption nexus led Greg Ardé (2020) to write his book *War Party, How the ANC’s political killings are breaking South Africa* in which he examined the connections that existed between KZN’s violent past and its murderous present (Mongale 2021:162).

**FIGURE 1:**
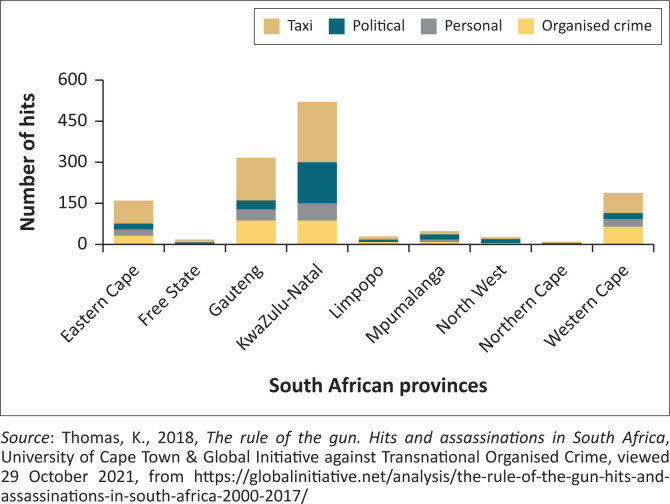
Assassinations per province and category by hitmen in South Africa: 2000–2017.

**FIGURE 2:**
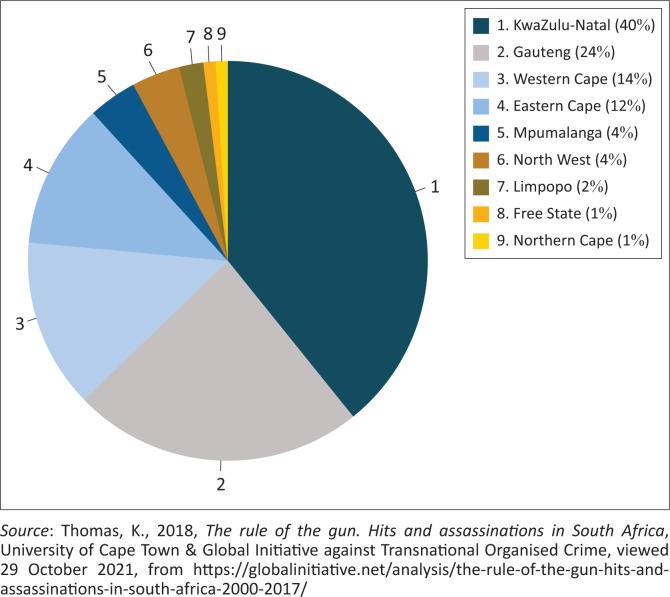
Hits and assassinations per province in South Africa for the period 2000–2017.

Greg Arde’s *War Party* (2020) reveals the way violence is linked to commerce in the essence of power, patronage and personal enrichment by those holding public office, who would use their power to crush those who stand in their way (Mongale 2021:162). KwaZulu-Natal under the ruling ANC and its mafia-style politics had become a risk to human security and the ANC’s intra-party violence in the province had connotations of a gangster state ‘where might was right and the big guns called the shots’ (Mongale 2021:162). As such, there is a prevalence of high contestation for political office in the ANC accompanied by violent conflict emanating from factions or individuals in the dominant ANC (Torgler & Frey 2013). Therefore, the violent political landscape has translated into insecurities throughout the province of KwaZulu-Natal because many people have lost their lives because of political assassinations. From 2000 to 2019, political violence in KZN took hold increasingly, especially targeted killings, using *izinkabi* (hitmen) (Shaw 2017:40–45), which increased from 2000 until 2017, as indicated in Figure 1. According to Figure 1, between 2000 and 2017, a total number of 1 373 cases of assassinations were recorded (Thomas 2018:10). Although the figure focuses on the period from 2000 to 2017, the article focuses on political violence in KZN from 2000 to 2019. When analysing the hits by provinces, Figure 2 indicates that the KwaZulu-Natal province accounted for 40% (i.e. 522 cases) of all recorded hits for the period 2000–2017, making the KZN province the highest contributor to political killings across South Africa (Thomas 2019:12)

Figure 2 provides data collected by Thomas (2018:11). The data reveal a number of trends by both province and category of activities, such as political killings, taxi industry targeted killings, personal killings and killings related to organised crime (Mongale 2022b:67). Based on the report by Thomas (2018:13), as published by the Global Initiative against Transnational Organised Crime, South Africa experienced a decline in criminal incidents in different categories. This was witnessed from 2000 to 2012, as indicated in Figure 2. However, between 2014 and 2017, there was an increase in the average annual incidents, which increased sharply, especially political violence, as seen in Figure 2 (Mongale 2022b:67). This could be attributed to the ruling party’s (ANC) dominance in South Africa’s political landscape and the administration of Jacob Zuma (2009–2018), whose tenure was characterised by violent competition for government positions and positions within the ANC structures (Mongale 2021:162), thus leading to an increase of politically related violence (Shaw & Thomas 2017:611).

The data in Figure 2 highlight that in South Africa, KZN Province accounted for 40% (i.e. 522 cases) of all hits recorded from 2000 to 2017 (Thomas 2018:11). Essentially, KZN, as indicated in Figure 2, stood out as the province that contributed largely to assassinations throughout the country. Although Thomas (2018:12) indicated that a breakdown of assassination incidents in KZN was consistent over longer periods, between 2000 and 2011, there was a general declining trend. However, there was a sharp increase in hits and assassinations, which started in 2012 and continued until 2017 (Mongale 2022b:63).

This increase in hits and assassinations could be linked to the presidency of Jacob Zuma, and during his tenure, political violence started to increase especially between members of the ruling party who fought and killed each other through factional lines, all in the name of competition for political offices such as councillorship (Thomas & Shaw 2017:602). It is important to notice that among the hits and assassinations, taxi industry incidents were the largest contributor to the overall trend of hits in KZN (Ardé 2020:118–119), followed by the most significant contributor to the trend, which was political incidents, and then organised criminal activities and hits relating to personal reasons, as highlighted in Figure 2 (Mongale 2022b:64). Sadly, these hits left many people traumatised, especially those whose family members were killed in their presence; meanwhile, other people were left in poverty especially after a family breadwinner was killed for political motives (Ardé 2020)

**FIGURE 3:**
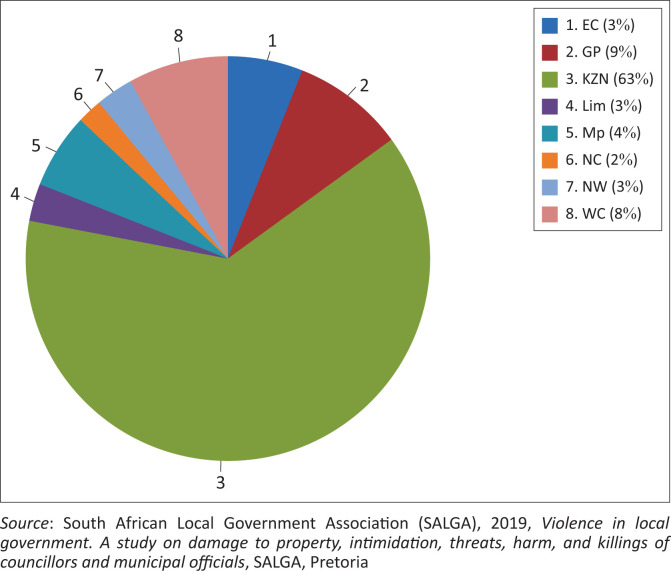
Assassinations of councillors per province.

The South African Communist Party (SACP) delegation led by Richard Mthembu testified before the Moerane Commission of Inquiry on 21 September 2016. According to the Tripartite Alliance member, the SACP, the continuation and the intensity of the killings within the Alliance could be traced back to the post-Polokwane and pre- and post-Local Government Elections of 2016 (Moerane Commission 2018:288). According to the SACP, the rapid increase in political violence and political killings within the ANC and its Alliance structures could be attributed to intra-party power struggles employed at leadership level (Mongale 2022b:68). Conversely, the witnessed political killings in KZN could be related to the killing of local government officials, who served because of unfair processes executed in the selection of councillors and candidates, and the subsequent deployment in municipal positions (Moerane Commission 2018:288). The former claims are supported by data provided in Figure 3, which highlight the percentages of assassinations per province in South Africa.

According to the data provided in Figure 3, municipal councillors were the officials most affected by political assassinations (SALGA 2019:39). The data presented in Figure 3 (a percentage breakdown of assassinations of councillors per province), confirms a total of 89 assassinations of councillors from 2000 to 2018. KwaZulu-Natal was the worst affected by assassinations, recording 63% of the hits from 2000 to 2018, as indicated in Figure 3 (Mongale 2022b:69). Gauteng province was in second spot with 9% of assassinations, followed by the Western Cape at 8%, and the Northern Cape and North-West being the provinces that were least affected with 2% and 3%, respectively (SALGA 2019:39). In short, as evident from Figure 3, data confirm that although assassinations of councillors remained a problem that affected all provinces, KZN was the province affected the most by targeted killings. (SALGA 2019:40)

The factionalism and intra-party tension seen within the ANC in KZN could also be attributed to the budget of the eThekwini Metro (Moerane Commission 2016:288). The budget was estimated to be more than 40 million (Ardé 2020:162) being one of South Africa’s metropolitan municipalities. Thus, it was alleged to be the main motivation for the intra-party conflict because the faction that emerged victorious would control the eThekwini Metro’s resources such as government tenders and contracts (Mongale 2022b:70). In addition, the other reason identified as a driver to control the Metro by one faction was that of having control over public transport tenders and housing projects (De Haas 2016:32).

In order to make sense of the politics of the eThekwini Region, the SACP testified (Moerane Commission 2018:289) that it was important to understand the dynamics of the link between the national power struggle and the local power struggle within the ruling ANC. The SACP further stated that divorcing the direct link between national politics and local politics would be a travesty of the political climate if the root cause of the problem were provincial leaders executing and carrying out the mandates, instructions and programmes of national leadership (Mongale 2022b:70).

Although the SACP conceded to having suffered heavy losses in its membership because of political killings in the KZN Province, to them what stood out was the incident of Intshanga (Moerane Commission 2018:289. The causality of the conflict between the ANC and SACP members came after members of the SACP won the 2016 local government elections against the seated ANC members (Makhaye & Mkhize 2017). Furthermore, members of the SACP postulated that the tension in Intshanga was instigated by a contestation for leadership in the eThekwini Region (Mongale 2022b:70). Meanwhile the SACP had a stronghold in Intshanga and showed their undivided support to James Nxumalo. Members of the SACP (who held dual membership with the ANC) were punished by the ANC in Intshanga as they were sidelined from the activities of the ANC (De Haas 2016:32). For instance, the SACP and/or ANC members supporting Nxumalo were denied the opportunity and the right to renew their ANC membership. Consequently, the majority of the SACP and/or ANC members in the Intshanga area were being marginalised by the ANC from actively participating in branch activities (Moerane Commission 2018:292).

According to the SACP, the tensions between the ANC and SACP reached boiling point when a meeting at the KwaNdokweni Stadium, organised by the SACP, was invaded by armed men who randomly fired shots, leading to the injury and death of many SACP attendants (Moerane Commission 2018:292). Following several attempts from the Alliance structures to reconcile the two factions, the ANC and the SACP of Intshanga received a colloquial welcome from the ANC leadership of the eThekwini Region (Mongale 2022b:71). Furthermore, the SACP held the view that the political killings involved the utilisation of state security companies, who subsequently served as personal security guards for some of the ANC politicians. The SACP also referred to the significant occurrence and practice of private security personnel being at the forefront in controlling political meetings on behalf of the ANC (Mongale 2022b:71). In reference to these assertions, various witnesses stated that there had been an increase in local government officials being surrounded by private security personnel (Moerane Commission 2018:294).

In the KZN Province, political violence and political killings took the form of inter-party and intra-party conflict (Mongale 2021: 162). These violent mechanisms began to increase in 2000; as Mongale (2022b:71) posits that after 2004, political violence in KZN province shifted its focus from inter-party to intra-party warfare. This came after the ANC’s dominance of the KwaZulu-Natal’s political arena, following the decline of support for the Inkatha Freedom Party (IFP), which had previously dominated the politics of KZN. In the same vein, we argue that the dominance of the ANC in KZN’s political domain after 2004 led to members of the ANC competing among themselves for political office. This form of competition resulted in individuals eliminating others as a means of improving their chances of standing for public office. As the saying goes ‘where there is competition, there is danger’ (SALGA 2019:68). These tendencies triggered a spree of violence whereby members of the same political party divided themselves into factions and competed with opposing factions (Mongale 2022b:71). The nexus between the ANC intra-party tensions and targeted killings served as risks to human security as they led to a vicious cycle of political assassinations, which negatively affected the security of people throughout the KZN Province. Political violence in the KZN province has resulted into a climate of fear and intimidation, especially where people are killed and no one is being held accountable (Mbayele 2022). The culture of impunity goes beyond political assassination because in other politically related violence such as violent protests, people have lost their properties due to vandalism, while their goods were looted, causing economic security, such as the case during the July 2021 riots in KZN and Gauteng (Mongale 2022a:8).

Violence that is employed for political motives serves as one of the leading threats to electoral democracies (SALGA 2019), particularly to emerging democracies. According to the Centre for the Study of Violence and Reconciliation (2018), political violence in contemporary democracies such as South Africa is influenced by poor socio-economic conditions such as poverty, unemployment and a lack of educational qualifications and inequality (Bruce 2015), which result in competition for controlling state economic resources (CSVR 2018). These socio-economic factors compel political leaders to erode political legitimacy and ultimately lead to discontent among various groups who are likely to resort to violence to seek concession from the government as a means of expressing their grievances (SALGA 2019:25). Often at times, these protest action leads to political instability. This coincided with the realities occurring in South Africa, whereby the country had a 27.6% unemployment rate, while the youth (aged 15–34 years) accounted for 63.4% of the total number of unemployed people in the country (Statistics South Africa 2019). Moreover, 55% of the population lived in poverty, including increasing inequalities at a 0.65 Gini coefficient (Statistics South Africa 2019). All these challenges of poverty, unemployment and inequality serve as threats to human security as highlighted in Table 1. These socio-economic challenges illustrated that human security in KZN is at risk because the perspective on human security advocates for a society that is free from ‘fear’ and free from ‘want’ (Alkire 2003).

In light of the foregoing, it is clear that factionalism has become a thorny issue within the ANC in KZN. As such, failure to address this crisis of factionalism has led to many political assassinations and fatal injuries, actions that are known to be disaster risks. Competition for resources and corruption from members of the ruling party in KZN have contributed largely to factional disputes and targeted killings, which have remained a threat to human security in the controversial province. For instance, because of corrupt practices, this has hampered the provision of basic services such as water and sanitation, electricity and infrastructure, all of which perpetuate unemployment, poverty and inequality (SALGA 2019:60–64). In this sense, the article with its objective was to reflect upon the crisis of factionalism and its impact on human security in KZN. The article recommends that in order for risk factors to be mitigated in KZN, the ruling party needs to unite and pursue similar goals. The existence of opposing sides within one organisation results in an unfriendly landscape for unity and cooperation, which will lead to animosity among opposing forces. Failure to mitigate the challenges of factionalism within the ruling party will exacerbate the risks to human security in KZN, resulting from intra-party warring.

## Conclusion

The advent of democracy in South Africa was characterised by inter-party political violence. This violence was seen between the two former liberation struggle movements, which turned into political parties. They were the two antagonists: the African National Congress (ANC) and the Inkatha Freedom Party (IFP). Political violence between these two parties was based on acquiring dominance of South Africa’s political landscape. However, following the dominance of KZN politics by the ANC post-2007, members of the same political party competed with each other for positions in both the government and party structures; thus, factional lines arose within one political party. Because of factional divisions, members of the ruling party resorted to killing and assassinating their fellow party members, especially those who competed for the same positions in the government and party structures. Often those who were outspoken about corruption in government were also eliminated through a bullet from a gun or intimidated not to take up government positions and roles in political party structures. This was the case with the assassination of the former African National Congress Youth League (ANCYL) secretary general Sindiso Magaqa. Magaqa was killed in 2017 in a drive by shooting along with two ANC colleagues in Umzimkhulu in KwaZulu-Natal. At the time of his death, he was elected as Ward 11 proportional representation councillor in Umzimkhulu municipality. He was assassinated for being outspoken about corruption in Umzimkhulu local municipality in KZN (Harper 2017). By intra-party tensions and targeted killings, many people lost their lives, others suffered from trauma caused by the killings, malicious bodily injuries, poverty (as a result of losing a breadwinner) and unemployment as a result of fearing to accept a job that could cost your life. Finally, failure by the ANC to resolve factional disputes will result in more threats to human security in KZN and this could spread throughout the country, as these killings have also occurred in provinces such as Mpumalanga, Eastern Cape, the North-West Province and Gauteng.
